# CD47 differentially regulates white and brown fat function

**DOI:** 10.1242/bio.056747

**Published:** 2020-12-16

**Authors:** Heather Norman-Burgdolf, Dong Li, Patrick Sullivan, Shuxia Wang

**Affiliations:** 1 Department of Pharmacology and Nutritional Sciences, University of Kentucky, Lexington, KY 40536, USA; 2 Department of Dietetics and Human Nutrition, University of Kentucky, Lexington, KY 40536, USA; 3 Department of Research and Development, Lexington VA Medical Center, Lexington KY 40502, USA; 4 Department of Neuroscience, University of Kentucky, Lexington, KY 40536, USA

**Keywords:** CD47, Brown fat, White fat, Lipolysis, Mitochondria

## Abstract

Mechanisms that enhance energy expenditure are attractive therapeutic targets for obesity. Previously we have demonstrated that mice lacking *cd47* are leaner, exhibit increased energy expenditure, and are protected against diet-induced obesity. In this study, we further defined the physiological role of *cd47* deficiency in regulating mitochondrial function and energy expenditure in both white and brown adipose tissue. We observed that *cd47* deficient mice (under normal chow diet) had comparable amount of white fat mass but reduced white adipocyte size as compared to wild-type mice. Subsequent *ex vivo* and *in vitro* studies suggest enhanced lipolysis, and not impaired lipogenesis or energy utilization, contributes to this phenotype. In contrast to white adipose tissue, there were no obvious morphological differences in brown adipose tissue between wild-type and knockout mice. However, mitochondria isolated from brown fat of *cd47* deficient mice had significantly higher rates of free fatty acid-mediated uncoupling. This suggests that enhanced fuel availability via white adipose tissue lipolysis may perpetuate elevated brown adipose tissue energy expenditure and contributes to the lean phenotype observed in *cd47* deficient mice.

## Abstract (precis)

**Summary:** Under physiological conditions, CD47 differentially regulates white fat and brown fat function. Through a coordinated manner, altered fuel availability via white adipose tissue lipolysis may influence brown adipose tissue energy expenditure and then body weight management.

## INTRODUCTION

Over the past thirty years, obesity has become a global health concern. Obesity is characterized by an excessive accumulation of body fat. Many obesity-associated comorbidities are a result of impaired adaptive mechanisms to manage increased lipid burden. Ideal therapeutic targets for obesity and its associated comorbidities are mechanisms that enhance lipid catabolism and mitochondrial function in response to increased lipid burden.

CD47 is a transmembrane cell receptor previously implicated in self-recognition and immune cell infiltration (Brown and Frazier, 2001; Matozaki et al., 2009; Oldenborg, 2013; Soto-Pantoja et al., 2015; Soto-Pantoja et al., 2013). CD47 activation negatively regulates intracellular cyclic guanosine monophosphate (cGMP) and cGMP-dependent protein kinase (PKG) activity (Isenberg et al., 2009 a; Isenberg et al., 2006; Ramanathan et al., 2011; Yao et al., 2011). cGMP/PKG signaling has been previously implicated as a necessary pathway for healthy adipocyte function and promoter of energy homeostasis. Downregulation of this specific signaling cascade has been observed in metabolically-compromised conditions including obesity, insulin resistance, and fatty liver disease (Handa et al., 2011; Rizzo et al., 2010; Tateya et al., 2011).

Previously, we identified a novel role of CD47 in a diet-induced obesity paradigm. We found that *cd47* deficiency protected mice from high fat diet induced obesity, which was attributed to the increased energy expenditure driven by brown adipose tissue (BAT) activity and these effects were not compromised under obese conditions (Maimaitiyiming et al., 2015). These studies further demonstrated elevated cGMP/PKG levels in BAT of *cd47-*deficient mice proposing a potential mechanism for enhanced energy expenditure (Maimaitiyiming et al., 2015). Interestingly, consistent with a previous report (Frazier et al., 2011), *cd47*-deficient mice exhibited a lean phenotype even under normal chow diet conditions. However, whether BAT activity drives this lean phenotype is not clear.

The aim of this manuscript is to further elucidate the mechanism contributing to a lean phenotype in *cd47*-deficient mice under physiological conditions. Findings in our current study have identified novel tissue-specific effects of CD47 on white and brown adipose tissue. *cd47*-deficient mice exhibit robust lipid turnover in white adipose tissue, which may provide a constant energy source for BAT energy expenditure. Additionally, we demonstrate that *cd47*-deficient mice have significant increases in UCP1 activity in BAT, which contributes to increased energy expenditure and the lean phenotype.

## RESULTS AND DISCUSSION

### *cd47*-deficient white adipose tissue depots contained more smaller size adipocytes, which was attributed to the increased lipolysis

**Fig. 1.**
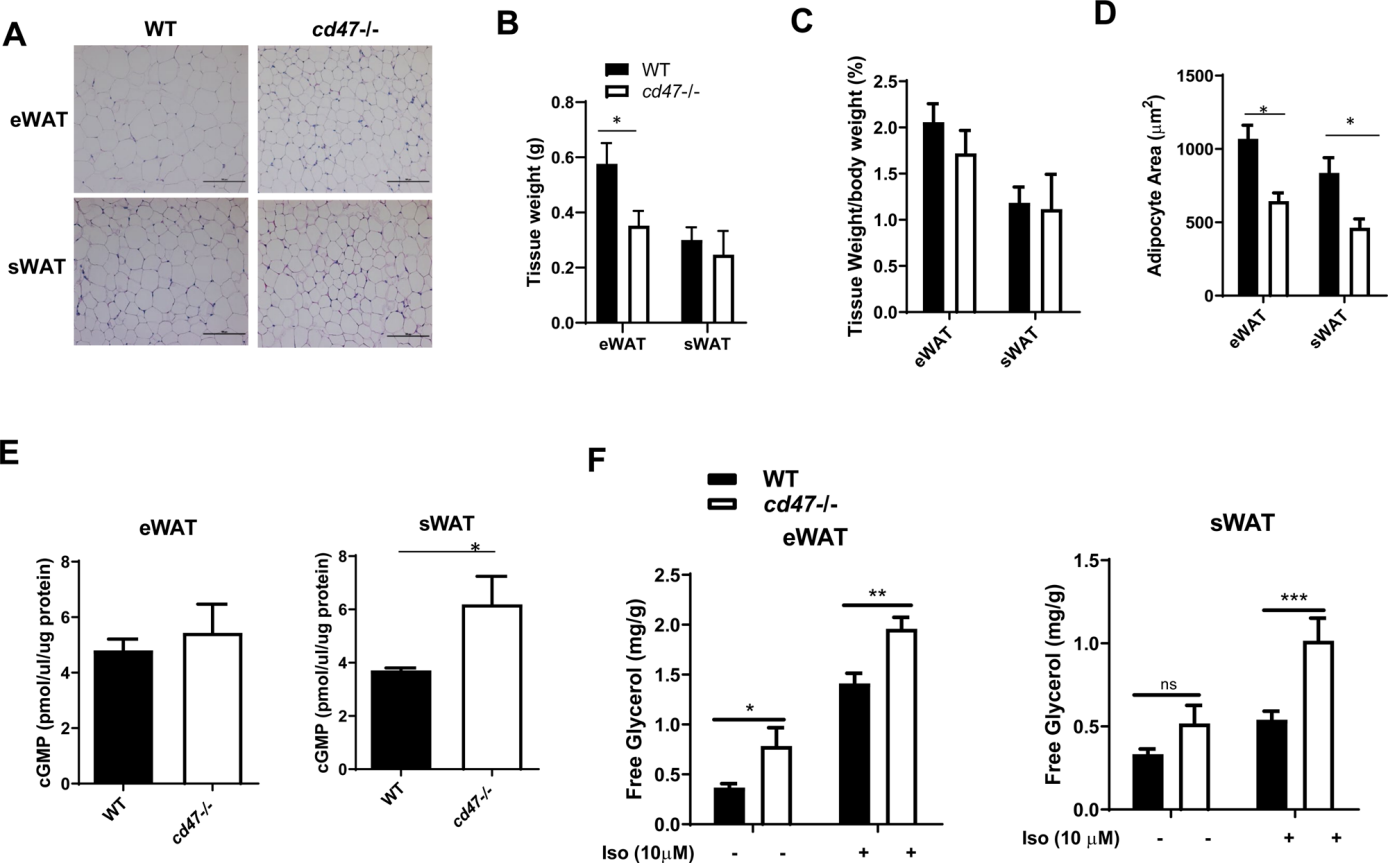
**Morphology and lipolysis of WAT in *cd47*-deficient mice and WT littermate controls.** (A) Representative images of H&E staining in eWAT and sWAT from *cd47*-deficient mice and WT littermate controls. Images obtained at 20x magnification. Scale bars: 100 µm. (B) Absolute weights of eWAT and sWAT and (C) relative eWAT and sWAT weight to body weight ratio are shown. (D) Adipocyte area quantified in eWAT and sWAT using image analysis software. (E) cGMP levels from eWAT and sWAT were analyzed. Data are presented as mean±s.e. (*n*=7 mice /group), *P<0.05. (E,F). Basal or 10 µM isoproterenol stimulated lipolysis of eWAT or sWAT was analyzed by measuring released glycerol in culture media (*n*=3 mice/group; all tissue samples from each mouse were triplicated). Two-way ANOVA with post-hoc analysis was used for statistical analysis. *P<0.05; **P<0.01; and ***P<0.001.

CD47 is a transmembrane cell receptor previously implicated in self-recognition and immune cell infiltration (Brown and Frazier, 2001; Oldenborg, 2013; Soto-Pantoja et al., 2015). However, previous work from our lab and others has shown that global *cd47* deficiency results in a lean phenotype (Fig. S1) and protection from the development of diet-induced obesity (Frazier et al., 2011; Maimaitiyiming et al., 2015). To further understand the regulatory role of CD47 on body composition and energy expenditure, the current studies were completed to determine what drives the lean phenotype in *cd47*-deficient mice under physiological conditions. Particularly, we examined the physiological role of *cd47* deficiency in regulating mitochondrial function and energy expenditure in both white and brown adipose tissue. White adipose tissue mass and adipocyte size was measured in both epididymal adipose tissue (eWAT) and subcutaneous adipose tissue (sWAT) depots from 8-week-old male *cd47*-deficient mice and wild-type (WT) mice. We found that *cd47* deficiency was associated with a significant reduction in adipocyte size (Fig. 1A,D). There was a significant reduction in eWAT mass in *cd47-/-* mice as compared to WT mice (Fig. 1B). However, there were no significant differences in relative eWAT or sWAT weight to body weight between WT and *cd47*-deficient mice (Fig. 1C).

It has been previously demonstrated that cGMP/PKG signaling promotes lipolysis by activating lipases critical for the hydrolysis of triglycerides in lipid droplets (Nikolic et al., 2011; Nishikimi et al., 2009; Sengen; et al., 2003). In addition, it has been well established that activation of CD47 [e.g. through its ligand thrombosponidn1 (TSP1)] suppresses cGMP/PKG signaling in a number of tissues including adipose tissue (Isenberg et al., 2009b; Maimaitiyiming et al., 2015; Rogers et al., 2014). cGMP signaling has been shown to be necessary for healthy adipocyte function including adipogenesis, lipolysis and mitochondria function (Chaves et al., 2011; Haas et al., 2009; Lafontan et al., 2008; Nikolic et al., 2011; Nishikimi et al., 2009; Sengenès et al., 2003). Interestingly we found that cGMP levels were significantly increased in sWAT from *cd47*−/− mice as compared to WT mice (Fig. 1E). eWAT had more TSP1 expression than sWAT (Fig. S2). *cd47* deletion significantly reduced TSP1 expression in sWAT but not in eWAT, which suggests that the differential expression of TSP1 could contribute to the differential cGMP elevation in sWAT versus eWAT. Consistently, we demonstrated that lack of CD47 expression enhanced isoproterenol stimulated glycerol release from both eWAT and sWAT depots (Fig. 1F). These findings suggest that CD47 is a potential regulator of the pathway(s) driving lipolysis.

### Adipogenesis and mitochondria function was not altered in *cd47* deficient white adipocytes as compared to WT cells

**Fig. 2.**
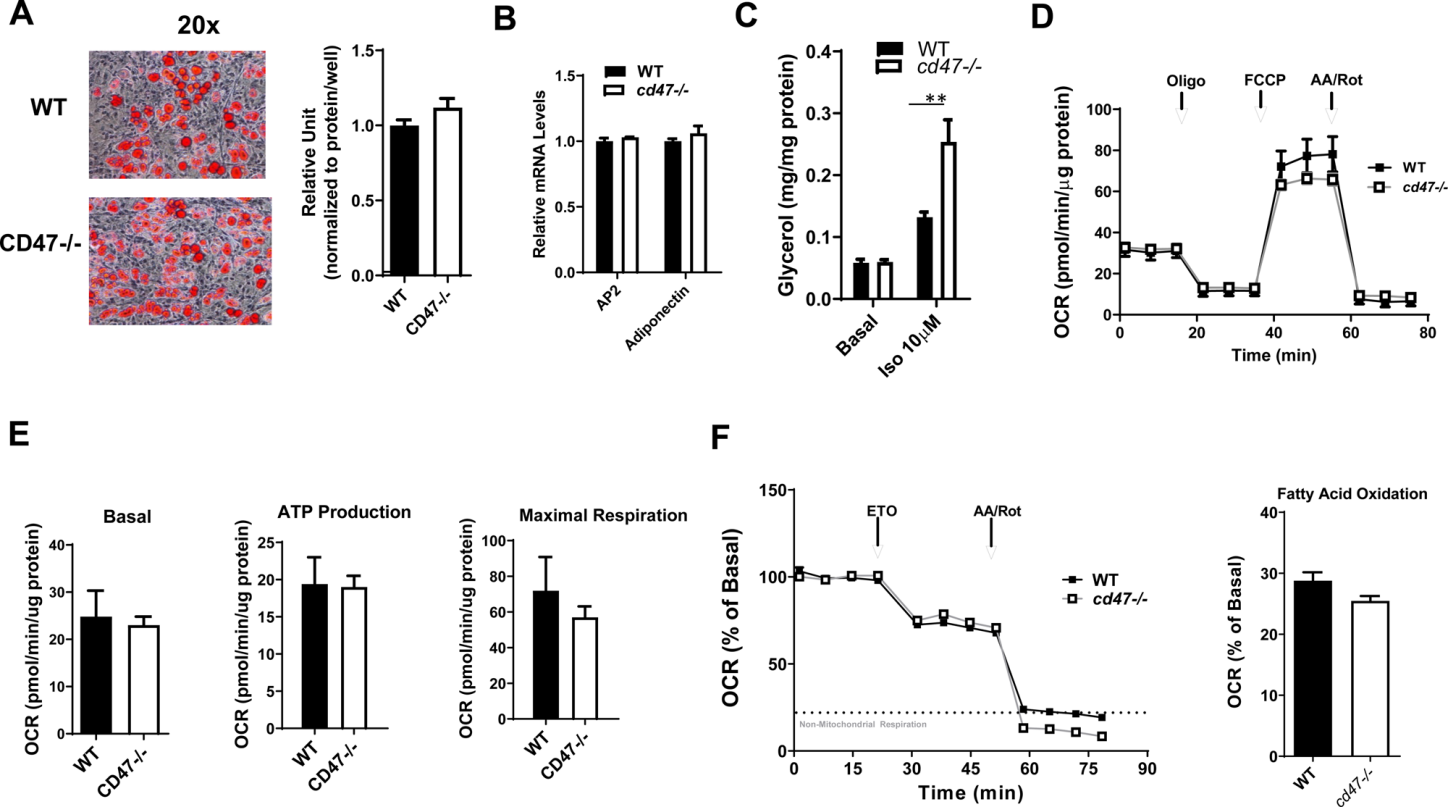
**Adipogenesis and mitochondrial function of primary white adipocytes isolated from *cd47*-deficient and WT mice.** (A) Primary white adipocytes were stained with Oil Red O to determine lipid accumulation on day 8 of differentiation and positive staining was quantified using a microplate reader. Microscope images were obtained at 20x magnification. Scale bar: 100 µm. (B) Real time PCR was used to determine the expression of AP2 and adiponectin gene from primary white adipocytes. (C) Basal or isoproterenol stimulated lipolysis in primary white adipocytes. (D) A basic mitochondrial stress test using the Seahorse XF96 Flux Analyzer was utilized to compare mitochondrial respiration in *cd47* deficient and WT primary adipocytes on day 8 of differentiation. (E) Basal respiration rates, ATP production, and maximal respiratory capacity were presented as pmol/min of oxygen in assay media. All values were normalized to protein/well. Five to seven replicates were used for each group. (F) Fatty acid oxidation (FAO) was determined by using Seahorse XF96 Flux Analyzer. Fatty acid β-oxidation was evaluated as maximum percentage of downregulation over baseline OCR after etomoxir injection. Data are presented as mean±s.e. (*n*=3 experiments). AA, antimycin A; OCR, oxygen consumption rate; rot, rotenone. **P<0.01.

In addition to lipolysis, several other mechanisms such as lipogenesis/lipid storage and fatty acid oxidation can regulate intracellular lipid levels within adipocytes (Calderon-Dominguez et al., 2016). Therefore, to determine whether *cd47* deficiency impairs triglyceride synthesis and storage in white adipocytes, stromal vascular fractions (SVF) from sWAT of *cd47* null or control mice were isolated and differentiated into white adipocytes. Our studies demonstrated no significant difference in lipid accumulation as indicated by Oil Red O staining of intracellular lipid from WT or *cd47* deficient primary white adipocytes (Fig. 2A,B). Consistent with *ex vivo* lipolysis data, *cd47*-deficient white adipocytes had increased isoproterenol stimulated lipolysis (Fig. 2C). Additionally, we determined whether there were any alterations of energy utilization between *cd47*-deficient cells and control cells. Mitochondrial respiration was assessed in *cd47*-deficient and control primary white adipocytes using the Seahorse XF96 Flux Analyzer (Agilent Technologies). Basal respiration, ATP production, and maximal respiratory capacity were determined by measuring oxygen consumption rates (OCR). *cd47*-deficient and control white adipocytes exhibited no significant difference in any mitochondrial function parameters (Fig. 2D,E). Fatty acid oxidation (FAO) assay was also performed in white adipocytes using Seahorse XF96 Flux Analyzer (Agilent Technologies). As shown in Fig. 2F, FAO capacity was similar between *cd47*-deficient and control white adipocytes. From these studies, we saw no differences in lipogenesis or mitochondrial function between genotypes *in vitro*. Taken together, these findings suggest that increased lipolysis might be a major contributor to the observed smaller adipocyte phenotype from *cd47* deficient white fat deport.

### Mitochondria from *cd47*-deficient BAT exhibited unique shapes and had enhanced FFA-mediated uncoupling as compared to wild-type BAT

**Fig. 3.**
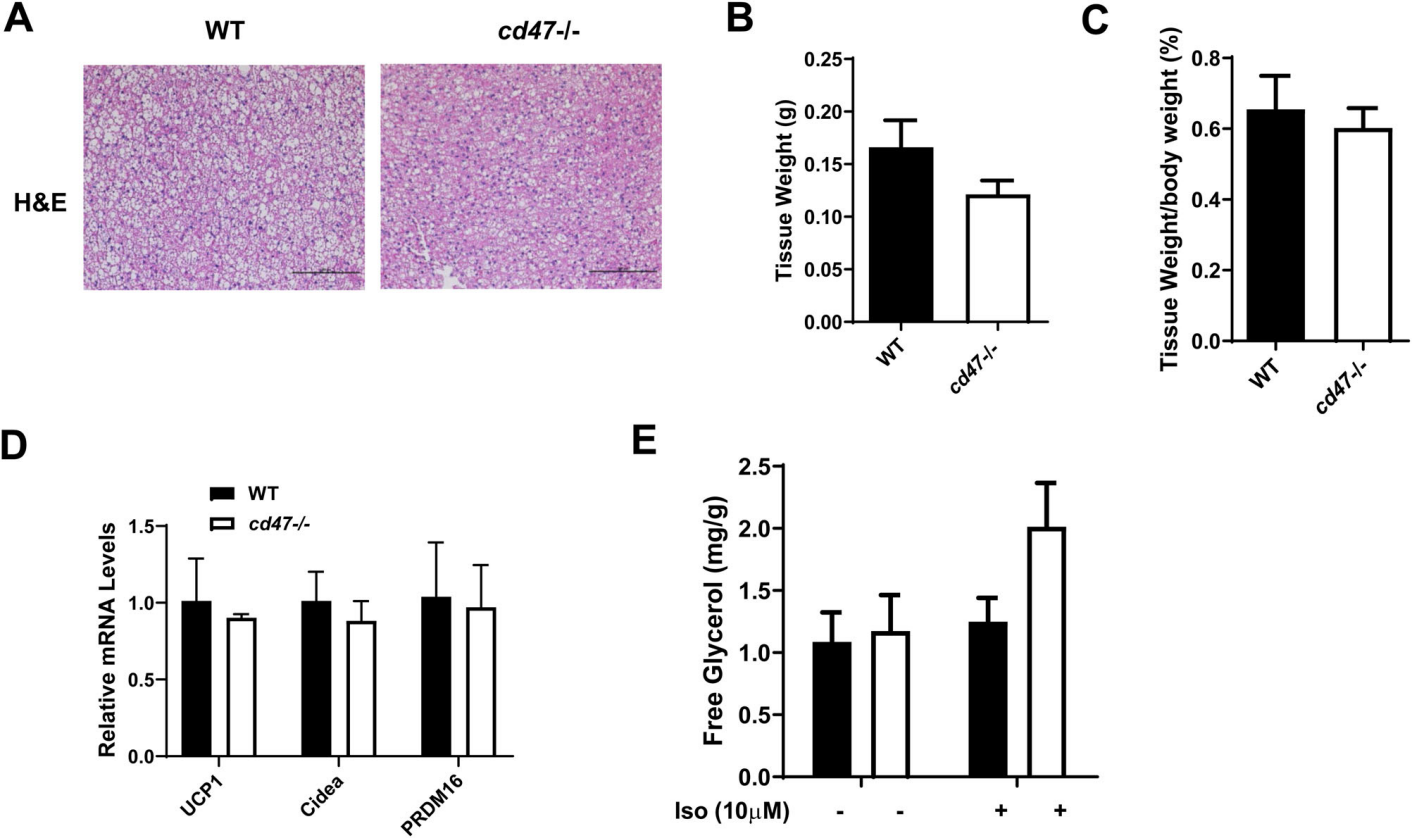
**Morphology and lipolysis of** **BAT** **in *cd47*-deficient and WT mice.** (A) Representative images of H&E staining in BAT from *cd47*-deficient mice and WT littermate controls. Images obtained at 20x magnification. Scale bars: 100 µm. (B) Absolute weight of BAT depots and (C) relative BAT weight to body weight ratio are shown (*n*=7/group). (D) Real-time PCR was used to determine the expression of BAT specific genes (e.g. UCP1, Cidea and PRDM16). (E) Lipolysis of BAT was analyzed. Data are presented as mean±s.e. (*n*=7 mice /group).

In the current studies, unique effects were observed in WAT of *cd47*-deficient mice compared with WT mice; therefore, BAT morphology and function was also examined. Unlike WAT, Hematoxylin and Eosin (H&E) staining exhibited no apparent morphological changes in BAT of *cd47*-deficient and WT mice (Fig. 3A) and no significant differences in BAT mass (Fig. 3B,C). In addition, expression levels of brown adipocyte specific genes such as UCP1, Cidea and PRDM16 in BAT were comparable between wild-type and *cd47* deficient mice (Fig. 3D). BAT *ex vivo* lipolysis rate was also similar between wild-type and *cd47*-/- mice (Fig. 3E). These data suggest that CD47 does not affect brown adipocyte adipogenesis or lipolysis.

**Fig. 4.**
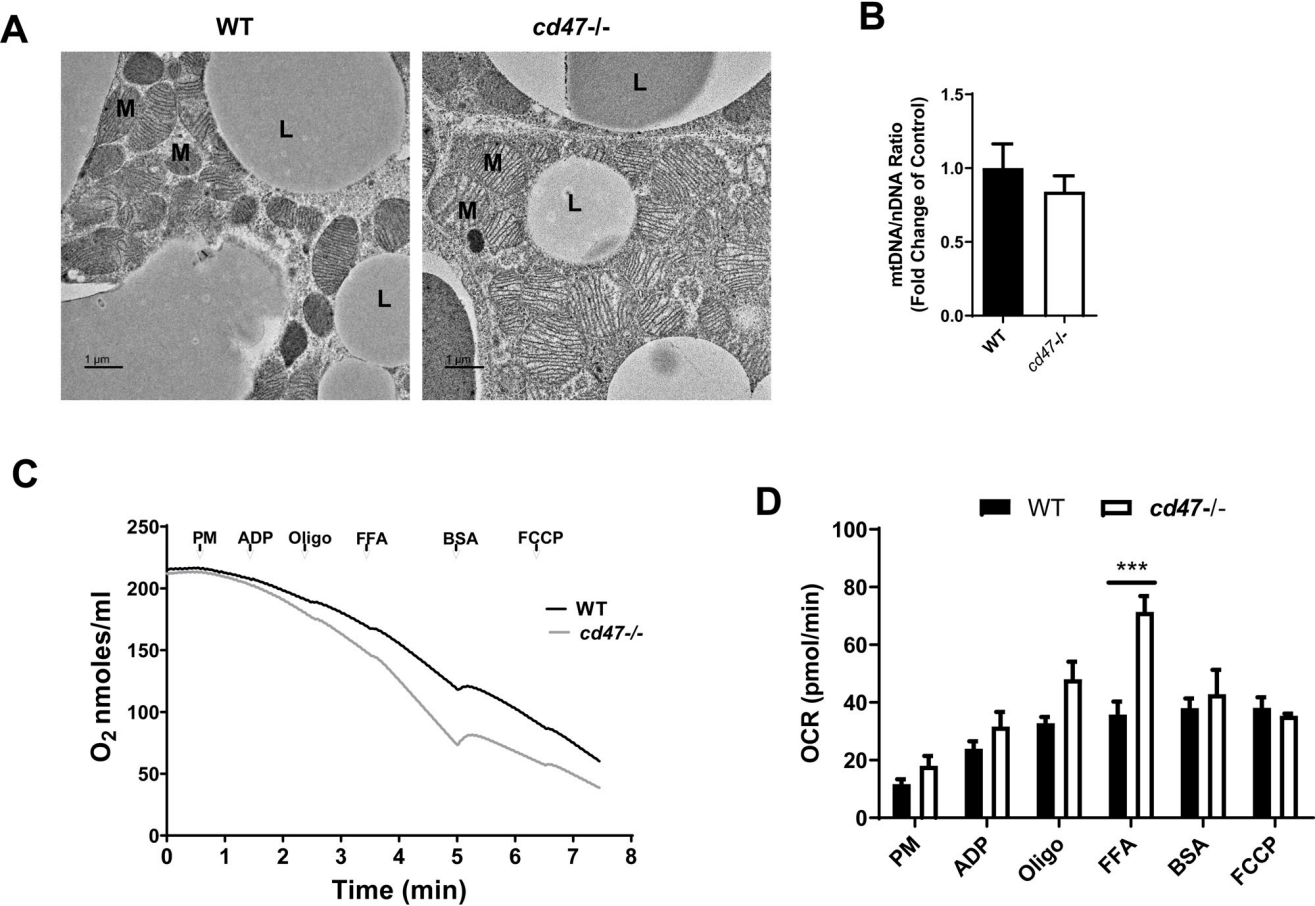
**Morphology and function of mitochondria from** **BAT** **in *cd47*-deficient mice and WT littermate controls.** (A) Representative images of transmission electron microscopy from BAT in *cd47*-deficient mice and WT littermate controls. Scale bars: 1 µm. L, lipid droplet; M, mitochondria. (B) mtDNA copy number in BAT was determined by real-time PCR. (C) Mitochondrial respiration (presented as oxygen consumption) was measured using a Clark-type oxygen electrode under sealed and isothermic conditions. Representative oxygraph traces are presented from isolated BAT mitochondria of *cd47*-deficient and WT mice. (D) After the addition of each compound, changes in oxygen consumption are quantified. Data are presented as mean±s.e. (*n*=4 mice/group), ***P<0.001. mtDNA, mitochondrial DNA; OCR, oxygen consumption rate; PM, pyruvate/malate; ADP, adenosine 5′-diphosphate; FFA, free fatty acid; Oligo, oligomycin; FCCP, carbonyl cyanide 4-(trifluoromethoxy)phenylhydrazone.

In BAT, free fatty acids (FFAs) have multiple physiological functions. Not only do FFAs serve as an efficient fuel source for increased energy requirements (Calderon-Dominguez et al., 2016), but also function as signaling molecules to activate UCP1 (Fedorenko et al., 2012) and to induce activation of multiple transcription factors implicated in energy expenditure (Townsend and Tseng, 2014; Wymann and Schneiter, 2008). Our lab previously reported that *cd47* deficiency may increase fatty acid translocation into mitochondria by fatty acid transporter CPT1b and drive the activation of UCP1 and subsequently increase energy expenditure in BAT (Maimaitiyiming et al., 2015). Interestingly, in the current study, the electronic microscope image showed that mitochondria from *cd47*−/− BAT had enlarged and elongated shape as compared to WT mice (Fig. 4A). However, mRNA levels of Drp1 (dynamin-1-like protein, an important regulator of mitochondria fission) were comparable between WT BAT and *cd47*−/− BAT (Fig. S3). CD47 has been shown to regulate the cellular localization of Drp1 in leukemic cells (Bras et al., 2007). Currently, whether there is any difference of cellular localization changes of Drp1 between WT BAT and *cd47*−/− BAT is unknown and warrants further investigation.

To determine whether the changes of mitochondria shape might lead to their altered functions (Campello and Scorrano, 2010), mitochondria were isolated from BAT of *cd47*-deficient and littermate control mice and treated with various compounds to examine mitochondrial bioenergetics, including UCP activity. Mitochondria isolated from *cd47*-deficient BAT demonstrated a significant increase in State IV and FFA-mediated uncoupling when compared with mitochondria isolated from BAT of WT mice (Fig. 4C,D). This indicates that *cd47*-deficient BAT mitochondria are adapted to consume more oxygen and generate more heat compared to WT in the presence of FFAs. Analysis of relative mitochondrial DNA (mtDNA) number indicates that increased mitochondrial biogenesis is not contributing to the significant changes in mitochondrial function in *cd47* deficient BAT (Fig. 4B).

In this study, we demonstrated that the metabolic function is regulated in a tissue-specific manner in the absence of CD47. For example, *cd47* deficiency enhances FFA-mediated uncoupling and energy expenditure in BAT; however, enhanced lipolysis is observed in WAT. While the studies included in this manuscript do not define what mechanisms contribute to tissue-specific CD47 function, several factors have been identified that may contribute to the unique effects of *cd47* deficiency in different tissues. Interestingly, we found that white fat tissue has higher TSP1 mRNA levels than brown fat tissue (Fig. S2). This suggests that the differences in CD47 function in white and brown adipose tissue might be secondary to the differential expression of its ligand-TSP1 in these tissues. In addition, CD47 has been shown to undergo post-transcriptional modifications. Recent studies have demonstrated that alternative 3′UTRs (untranslated regions) can differentially regulate CD47 cellular localization and functions (Berkovits and Mayr, 2015). To date, specific CD47 isoform expression in adipose tissue has not been determined. CD47 may also undergo post-translational modifications, including glycosylation in a tissue-specific manner (Brown and Frazier, 2001). Lateral associations and intramembrane cell receptor complexes including CD47 may differ between cell types (Kaur et al., 2010). Finally, varying expression levels of CD47 in different tissues as well as the concentration of the specific CD47 ligand/binding partner will determine activity levels and function. Tissue-specific isoform expression, structural changes, and various lateral/ligand interactions may suggest unique functional roles of CD47.

Together, our data suggests that CD47-mediated differential regulation of white and brown fat function contributes to the metabolic phenotype observed in the current study. However, to demonstrate definitively the effect of adipocyte derived CD47 on energy metabolism, tissue specific *cd47*-deficient mice are required in future studies.

## MATERIALS AND METHODS

### Animal studies

Eight-week old male *cd47* deficient mice (C57BL6/J background from Jackson Laboratories) and age-matched WT littermate controls were used for all studies. Experiments involving mice conformed to the National Institutes of Health Guide for the Care and Use of Laboratory Animals and were approved by the University of Kentucky Institutional Animal Care and Use Committee.

### Tissue histology

Adipose tissue depots were embedded in paraffin, sectioned at 4 μm, and stained with H&E by the University of Kentucky Pathology Core services from the Center of Research in Obesity and Cardiovascular Disease by standard procedure. All images were acquired with a Nikon Eclipse 55i microscope at 20× objective. White adipocyte area was quantified using the Object Count feature of Nikon NIS Elements BR software (Melville, NY, USA). Forty adipocytes were measured per section (*n*=3 sections/mouse) from mice in each group (*n*=3 mice/group).

### cGMP Measurements

cGMP levels in white fat tissue from wild type or *cd47*-deficient mice were measured by using the cGMP Direct Immunoassay Kit (Colorimetric) from Biovision (Milpitas, CA, USA). Frozen tissues were homogenized and the supernatant was collected. cGMP levels in the supernatant were measured and calculated based on the cGMP standard curve following the instruction manual.

### Lipolysis assays

Subcutaneous adipose tissue (SAT), epididymal adipose tissue (EAT), and BAT were excised from 8-week-old male *cd47*-deficient and WT littermate control mice after a 6-h fast. Tissue was weighed and kept in ice cold PBS until all tissue was collected. Adipose tissue (80–180 mg) was placed in one well of a 24-well plate (*n*=3 mice/group; all tissue samples from each mouse were triplicated). Tissues were serum starved in Dulbecco's Modified Eagle Medium (DMEM, Gibco, Carlsbad, CA, USA) supplemented with 1% fatty acid-free bovine serum albumin (BSA) for 1 h at room temperature (RT). Tissue was then treated with Krebs Ringer Buffer (125 mM NaCl, 5 mM KCl, 1.8 mM CaCl_2_, 2.6 mM MgSO_4_, 5 mM HEPES, pH adjusted to 7.2) in the presence of vehicle or isoproterenol (10 µM, Sigma-Aldrich, St. Louis, MO, USA) as previously described (Guo et al., 2013). After 2 h, media was collected to measure glycerol release with the Free Glycerol Reagent and appropriate standards (Sigma-Aldrich, St. Louis, MO, USA). Values were normalized to gram tissue weight (*n*=3 animals/group and *n*=3 experimental replicates/animal).

### Adipocyte differentiation

SVF was isolated from subcutaneous WAT as previously described (Gupta et al., 2010). Briefly, WAT was excised from 8-week-old male *cd47*-deficient and WT littermate controls, minced, and digested in Ca^2+^/Mg^2+^-free HBSS supplemented with 0.1% collagenase type II (Catalog No. C6885, Sigma-Aldrich, St. Louis, MO, USA) for 45 min in a 37°C shaking water bath. After digestion, cells were centrifuged at 700×***g*** for 10 min at 4°C and the supernatant including the adipocyte layer was removed. The pellet was digested a second time with collagenase type II digestion buffer. Cells were then filtered through a 100 μm cell strainer and centrifuged again at 400×***g*** for 5 min at 4°C. Cells were suspended in culture media Dulbecco's Modified Eagle Medium (DMEM, Gibco, Carlsbad, CA, USA) supplemented with 10% FBS and 1% penicillin-streptomycin. The SVF was used for differentiation and mitochondrial respiration studies. Cell differentiation was induced as previously described (Sui et al., 2014) by high glucose-DMEM supplemented with insulin (1.7 µM), 3-isobutyl-1-methylxanthine (IBMX; 0.5 mM), dexamethasone (1 µM), and 10% FBS. After induction, cells were cultured in high glucose-DMEM supplemented with insulin (1.7 µM) and 10% FBS until day 8 of differentiation (Sui et al., 2014).

### *In vitro* Oil Red O staining

Mature adipocytes were fixed with 4% paraformaldehyde and subsequently washed with PBS and freshly prepared 60% isopropanol in water. Cells were stained with Oil Red O (three volumes of 0.5% Oil Red O in 100% isopropanol with two volumes distilled water) for 30 min and washed with water. Images were acquired with a Nikon Edipse 55i microscope. To quantify lipid accumulation, extracted Oil Red O samples (in 100% isopropanol) were loaded in triplicate into a microplate and absorbance was read at 500 nm (Townsend et al., 2013). Absorbance values were normalized to protein content per well determined by BCA assay and presented as fold change over control.

### Cellular OCR using XF96 extracellular flux analyzer

Adipocyte OCR were measured with the Seahorse Biosciences XF96 Extracellular Flux Analyzer (Agilent Technologies) and used to determine basic parameters of mitochondrial function including basal respiration, ATP-linked respiration, and maximal respiratory capacity. Cell density of mature adipocytes for XF96 plates (Agilent Technologies) were 1×10^4^ cells/well. Cells were seeded 5 h prior to the assay to ensure adequate adherence to the plate. Using the protocol provided by Seahorse Biosciences and as previously described (Zaytseva et al., 2015), cells were treated with three different compounds and the OCR was measured before and after each treatment. Experimental compounds were prepared in assay medium including DMEM media with 10 mM glucose, 1 mM pyruvate and 2 mM glutamine. First, cells were treated with oligomycin (1 µM), which blunts ATP synthesis by blocking ATP synthase. Second, cells were treated with carbonyl cyanide-4-(trifluoromethoxy) phenylhydrazone (FCCP, 2 µM) which uncouples the electron transport chain and induces high oxygen consumption and energy expenditure without generating ATP. Finally, cells were treated with a combination of rotenone (complex I Inhibitor) and antimycin A (complex III inhibitor) (both 1 µM). This combination inhibits all mitochondrial respiration and allows the non-mitochondrial respiration of the cell to be measured. Analysis of data was completed with XFe Wave Software (Agilent Technologies). All values were normalized to cellular protein levels which were determined by a modified BCA Assay (Pierce) based off the manufacturer's protocol.

FAO test was also determined by using the Seahorse Biosciences XF96 Extracellular Flux Analyzer (Agilent Technologies). Cells were seeded on Seahorse XF-96 plates at a density of 1×10^4^ cells/well and incubated for 5 h in culture media. To assess FAO, 1 h prior to the assay, culture media was replaced with 170 μl of FAO assay media (XF Base DMEM Medium supplemented with 2 mM glucose and 0.5 mM Carnitine, pH 7.4). During the assay, 30 μl of 1 mM Palmitate-BSA (XF palmitate–BSA, Agilent Technology) was added to each well and the final concentration of palmitate was 0.15 mM. OCR was measured at baseline as well as after sequential injection of etomoxir (40 μM) and a combination of Rotenone and antimycin A (1 μM). OCR were expressed as a percentage of the baseline measurement. Fatty acid β-oxidation was evaluated as maximum percentage of downregulation over baseline after etomoxir injection.

### Assessment of isolated mitochondrial bioenergetics

BAT mitochondria were isolated using differential centrifugation with some modifications to the previously described methods (Sullivan et al., 2003; Sullivan et al., 2000). Briefly, 500–800 mg adipose tissues were excised, minced with a blade, and placed in isolation buffer with EGTA (215 mM mannitol, 75 mM sucrose, 0.1% BSA, 20 mM HEPES, 1 mM EGTA, pH adjusted to 7.2 with KOH). Tissues were mechanically homogenized at 300 rpm in 4 ml ice cold isolation buffer with EGTA. The homogenate was centrifuged twice at 1300×***g*** for 3 min in a 2 ml centrifuge tube at 4°C. Each supernatant fraction was collected in separate tubes and topped off with isolation buffer with EGTA and finally centrifuged at 13,000×***g*** for 10 min. The mitochondrial pellet was then suspended in 1 mL isolation buffer without EGTA and centrifuged for 10 min at 10,000×***g***. Finally, the mitochondrial pellet was resuspended in 30–50 µl isolation buffer without EGTA and stored on ice until the time of assay. The protein concentration was determined using the BCA assay kit (Pierce, Waltham, MA, USA) following the manufacturer's protocol.

Mitochondrial respiration was assessed using a Clark-type oxygen electrode (Hansatech Instruments), in a sealed, thermostatically controlled (37°C), and continuously stirred chamber as described previously (Sullivan et al., 2003; Sullivan et al., 2004). Mitochondria were added to the chamber to yield a final protein concentration of 50 µg in 250 µl. State II respiration was initiated by the addition of oxidative substrates, pyruvate and malate (5 mM and 2.5 mM, respectively). State III respiration was initiated by the addition of 120 nmol ADP followed by the addition of oligomycin (1 µM) to induce state IV respiration. UCP-mediated proton conductance was measured as increased free fatty acid (60 µM linoleic acid) induced respiration (Sullivan et al., 2003; Sullivan et al., 2004), followed by recoupling of the mitochondria by sequestration of FFA with the addition of BSA to a final concentration of 3%. Finally, mitochondria were treated with FCCP (1 µM) to allow for quantification of complex I driven, maximal electron transport.

### mtDNA copy number

mtDNA copy number in BAT was determined as previously described (Maimaitiyiming et al., 2015). The relative copy number was determined by real-time PCR and normalized to nuclear DNA (28 s). Primer sequences for mouse mtDNA were forward: 5′-CCG-CAA-GGG-AAA-GAT-GAA-AGA-C-3′ and reverse: 5′-TCG-TTT-GGT-TTC-GGG-GTT-TC-3′. Sequences for mouse 28 s were forward: 5′-GCC-AGC-CTC-TCC-TGA-TTT-TAG-TGT-3′ and reverse: 5′-GGG-AAC-ACA-AAA-GAC-CTC-TTC-TGG-3′.

### Real-time quantitative PCR

Total RNA from frozen BAT or cells was extracted using RNeasy Mini Kit (Qiagen, USA). RNA was reverse transcribed to cDNA by High Capacity cDNA Reverse Transcription Kit (Invitrogen, Carlsband, CA, USA). Real-time quantitative PCR was performed on a MyiQ Real-time PCR Thermal Cycler (Bio-Rad) with SYBR Green PCR Master Kit (Qiagen, Valencia, CA, USA). Relative mRNA expression was calculated using the MyiQ system software as previous reported (Li et al., 2011) and normalized to β-actin mRNA levels. All primer sequences utilized in this study are listed as below: AP2: forward AAGCCCACTCCCACTTCTTT, reverse TCACCTGGAAGACAGCTCCT; Adiponectin: forward AACATTCCGGGACTCTAC T, reverse TACTGGTCGTAGGTGAAGAG; UCP1: forward (5′ to 3′) ACTGCCACACCTCCAGTCATT, reverse (5′ to 3′) CTTTGCCTCACTCAGGATTGG; Cidea: forward AGGGACAACACGCATTTC, reverse GTAGGACACCGAGTACATCT; PRDM16: forward CACAAGACATCTGAGGACAC, reverse CTCGTGTTCGTGCTTCTT; TSP1: forward TGTGGAAACAAGTCACCCAGTCCT, reverse TTTCCTGTGTGCCACAGTGCATTC; Drp1: forward ACCGGGAATGACCAAAGTACCTGT, reverse ACGGCGAGGATAATGGAATTGGGA.

### Transmission electron microscopy

For ultrastructural analysis of mitochondria, fragments of the BAT obtained from WT and *cd47*-deficient mice were dissected and fixed with 2.5% glutaraldehyde in cacodilate buffer 0.1 M (pH 7.2). The samples were shipped to August University Electron Microscopy Core Facility for further processing and imaging.

### Statistical analysis

Statistical analysis was performed using Prism version 8.0.2 (GraphPad Software, San Diego, CA, USA). Data are expressed as mean values±s.e. Statistical significance between two groups was determined using two-tailed Student's *t*-test. One-way ANOVA followed by Bonferroni's multiple comparisons test or two-way ANOVA followed by Tukey's multiple comparisons test was applied for multi-group comparisons. *P*-values of less than 0.05 were considered to be significant.

## Supplementary Material

Supplementary information
