# Supplementary material for: CD47 differentially regulates white and brown fat function
Source: Biol Open. 2020 Dec 16;9(12):bio056747. doi: 10.1242/bio.056747 (PMC7758621; doi:10.1242/bio.056747)
Supplement: Supplementary information [file biolopen-9-056747-s1.pdf]

Biology Open (2020): doi:10.1242/bio.056747: Supplementary information

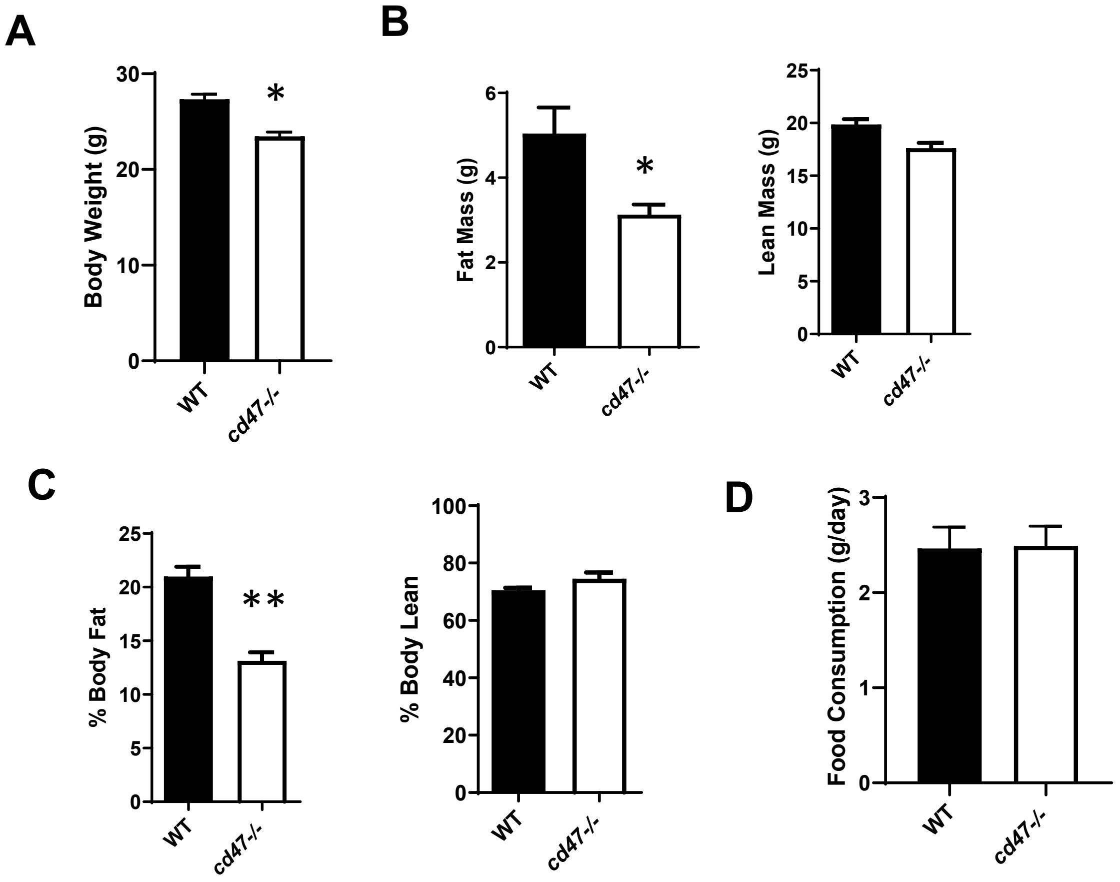


**Fig. S1**. Body weight (A), body composition determined by EchoMRI including fat mass and lean mass (B) and percent of fat mass and lean mass to body weight (C), and daily food consumption (D) from male WT and *cd47* deficient mice under normal chow diet. Data are represented as mean ± SE (N=7 mice/group). *P<0.05 vs. WT; **P<0.01 vs. WT.

Biology Open • Supplementary information

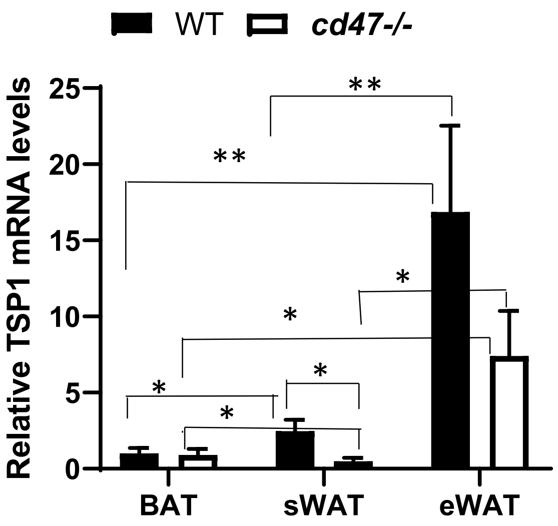


**Fig. S2**. Expression of TSP1 in different fat depots from WT or *cd47* deficient mice was determined by real-time PCR and normalized to β-actin levels. Data are represented as mean ± SE (N=7 mice/group). *P<0.05; **P<0.01.

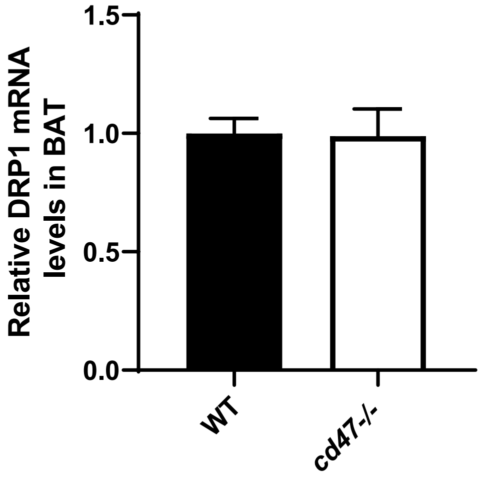


**Fig. S3.** Expression of DRP1 in BAT from WT or *cd47* deficient mice was determined by real-time PCR and normalized to β-actin levels. Data are represented as mean ± SE (N=7 mice/group).
